# The intralaminar thalamus—an expressway linking visual stimuli to circuits determining agency and action selection

**DOI:** 10.3389/fnbeh.2014.00115

**Published:** 2014-04-02

**Authors:** Simon D. Fisher, John N. J. Reynolds

**Affiliations:** Department of Anatomy, Brain Health Research Centre, School of Medical Sciences, University of OtagoDunedin, Otago, New Zealand

**Keywords:** intralaminar, basal ganglia, learning, plasticity, sensory, action selection, agency

## Abstract

Anatomical investigations have revealed connections between the intralaminar thalamic nuclei and areas such as the superior colliculus (SC) that receive short latency input from visual and auditory primary sensory areas. The intralaminar nuclei in turn project to the major input nucleus of the basal ganglia, the striatum, providing this nucleus with a source of subcortical excitatory input. Together with a converging input from the cerebral cortex, and a neuromodulatory dopaminergic input from the midbrain, the components previously found necessary for reinforcement learning in the basal ganglia are present. With this intralaminar sensory input, the basal ganglia are thought to play a primary role in determining what aspect of an organism’s own behavior has caused salient environmental changes. Additionally, subcortical loops through thalamic and basal ganglia nuclei are proposed to play a critical role in action selection. In this mini review we will consider the anatomical and physiological evidence underlying the existence of these circuits. We will propose how the circuits interact to modulate basal ganglia output and solve common behavioral learning problems of agency determination and action selection.

## Introduction

An early view of the function of the intralaminar thalamus attached a nonspecific role to these nuclei in propagating reticular arousal signals (Steriade and Glenn, 1982; Groenewegen and Berendse, 1994). Recent anatomical and electrophysiological evidence has, however, painted the intralaminar nuclei in a much more behaviorally influential light. Through functional relationships with primary sensory areas and the basal ganglia, these nuclei have emerged as key contributors to circuits that underlie behavioral learning and selection processes.

The foundation for this new role emanated from early anatomical descriptions of ascending projections from the midbrain superior colliculus (SC) to the thalamic nuclei. A general division of the SC was proposed by Harting et al. (1973) between the superficial visual layers, which project to the thalamic visual centers described at that time, and the deep motor and arousal layers, which project to the non-visual thalamic intralaminar nuclei, and other subcortical motor and arousal areas. The intralaminar nuclei had in turn been found by Cowan and Powell (1956) to contribute a major projection to the striatum, the primary input nucleus of the basal ganglia.

A primary characteristic of the SC is its particular sensitivity to the appearance of salient stimuli, such as novel objects and loud or bright events (Wurtz and Albano, 1980; Sparks, 1986). It responds to such stimuli at very short latency, preceding any gaze shift intended to bring a newly appearing stimulus into view, and therefore is an ideal candidate to provide early sensory signals to subcortical processing units. The intralaminar nuclei have been recently shown to play a critical role in allowing this short-latency signal to gain access to circuits in the basal ganglia.

Recent studies have demonstrated that the appearance of a salient sensory event will lead to a temporal convergence in the striatum of a sensory signal via the intralaminar nuclei (Schulz et al., 2009) and a dopaminergic signal (Dommett et al., 2005). These inputs have been considered in previous work as necessary substrates for changes in synaptic efficacy at corticostriatal synapses, a cellular mechanism thought to underlie reinforcement learning in the basal ganglia (Reynolds and Wickens, 2002). One function that relies on reinforcement learning processes at short-latency is the ability to determine agency—to discover causal relationships in our world between actions and their outcomes (Redgrave et al., 2011). Another function that exploits the convergence of short-latency signals, via proposed tecto-thalamo-striatal loops, is action selection (McHaffie et al., 2005).

In this mini review we will précis the anatomical and physiological evidence underlying the existence of subcortical loops between the SC, intralaminar and striatal circuits. We will propose how changes in synaptic efficacy via the short-latency convergence of critical learning signals can resolve the problems of agency determination and behavioral selection.

## Anatomy

The mammalian SC is a laminated midbrain structure consisting of three layers: the superficial, intermediate and the deep layers. These can be subdivided, based on cytoarchitecture, into a total of seven layers, alternating between cell-rich and fiber-rich layers (Kanaseki and Sprague, 1974). See May (2006) for an extensive cross-species review of the structure.

The superficial layers are primarily visual, receiving direct retinal input together with indirect visual information from areas of the visual cortex. These layers project to visual thalamic nuclei, including the lateral geniculate and pulvinar nuclei, and also down to the deep layers of the SC (Harting et al., 1992; May, 2006). The deep layers are multimodal and incorporate inputs from somatosensory and auditory sources, together with non-sensory modulatory inputs from cortical and subcortical areas (Gaese and Johnen, 2000). Anatomical studies in the rat reveal that the intermediate and deep SC layers send glutamatergic projections to both rostral and caudal intralaminar thalamic nuclei (Krout et al., 2001; Van der Werf et al., 2002). The caudal intralaminar nuclei comprise the center median and parafascicular (CM/Pf) nuclei in primates. In rodents, the lateral portion of the Pf is homologous to the primate CM (Groenewegen and Berendse, 1994; Van der Werf et al., 2002). The rostral intralaminar nuclei comprise the central medial, paracentral and central lateral nuclei. It is noteworthy that the thalamic nuclei targeted by the SC are those that provide the primary thalamic input to the basal ganglia (Takada et al., 1985; Féger et al., 1994).

**Figure 1:**
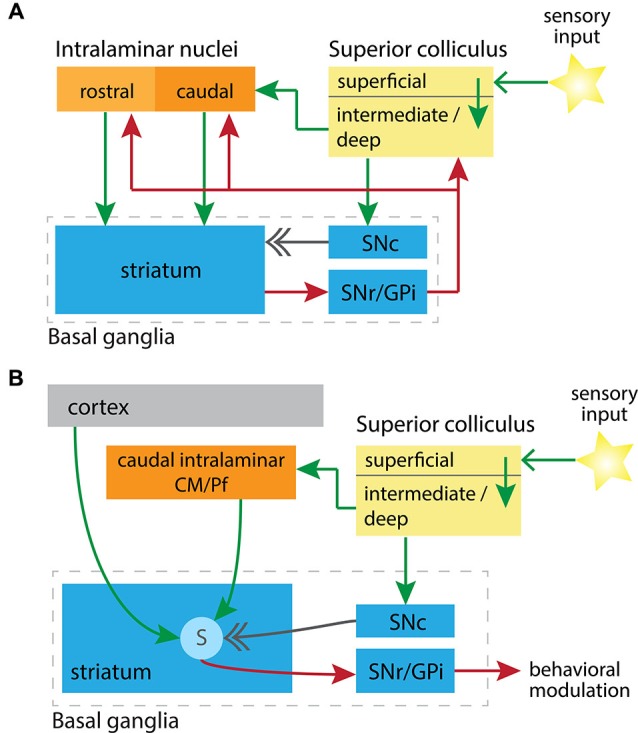
Circuit diagrams illustrating **(A)** the synaptic relationships between key subcortical nuclei, and **(B)** the convergence in the striatum of learning signals from these nuclei. In both diagrams excitatory projections are green, inhibitory are red, and the neuromodulatory dopaminergic projection is in gray. **(A)** Putative subcortical loops are formed between the SC, rostral or caudal intralaminar nuclei, and through the basal ganglia to return to the SC. SNc, substantia nigra pars compacta; SNr/GPi, substantia nigra pars reticulata and globus pallidus internal segment. **(B)** Convergence onto striatal spiny projection neurons (labeled “S”) of a short-latency sensory signal from the SC, and a related short-latency dopaminergic signal from the SNc. When this conjunction is aligned with cortical input to spiny neurons, representing motor efferent copy, then the factors previously identified as necessary for potentiation of corticostriatal synapses are present (Reynolds and Wickens, 2002). CM/Pf, center median and parafascicular nuclei.

The major target of the intralaminar nuclei, previously thought to be cortical, is the striatum (Parent et al., 1983; Smith and Parent, 1986; Sadikot et al., 1992). Via synapses onto striatal spiny neurons, the intralaminar nuclei modify the output from the striatum through either the direct or indirect pathway to the principal output nuclei of the basal ganglia: the substantia nigra pars reticulata (SNr) and the internal globus pallidus (GPi), respectively. The basal ganglia outputs return direct projections to the caudal intralaminar CM/Pf complex from the GPi (Kuo and Carpenter, 1973; DeVito and Anderson, 1982; Parent et al., 1983; Sidibé et al., 1997) and the SNr (de las Heras et al., 1998; Sidibé et al., 2002; Tsumori et al., 2003). The basal ganglia output nuclei also project to the midbrain, including the SC (Redgrave et al., 1992), and indirectly via the thalamus to cortical and limbic regions from which basal ganglia input originated (Alexander et al., 1986; Parent and Hazrati, 1995; Smith et al., 2004). See Figure 1A for an abstracted high-level view of these structures and relations.

The subcortical structures reviewed here, including the SC (Stein and Gaither, 1981), thalamic nuclei (Butler, 1994), and basal ganglia (Reiner et al., 1998), are all evolutionarily ancient, and appear well-conserved in vertebrates. It has been proposed that these highly interconnected subcortical structures evolved to function together for survival in primitive organisms, prior to the emergence of the cortex in higher order vertebrates (McHaffie et al., 2005).

## Reinforcement learning

One of the first functional roles ascribed to the SC was to direct eye gaze towards or away from salient events, owing to its sensitivity to novel objects and loud or bright stimuli (Wurtz and Albano, 1980; Sparks, 1986). SC neurons respond with short-latency bursts of activity, tightly coupled to stimulus onset (Jay and Sparks, 1987). Putative spiny neurons in the dorsal striatum are also activated by salient visual stimuli, with a short-latency (100–150 ms) response that typically precedes the gaze shift time required (150–200 ms) to foveate to a salient visual event (Sparks, 1986; Hikosaka et al., 1989). This suggests that the responses of the SC and dorsal striatal neurons are involved in early, pre-foveation, processing of important environmental events.

It was recently shown that the short-latency striatal response is mediated by a pathway from the SC to the striatum via the intralaminar nuclei (Figure 1B). Schulz et al. (2009) found that light flashes presented to a rat’s eye drove dorsal striatal spiny neurons to their depolarized “up state” within a latency of approximately 115 ms. This visually-evoked response was abolished with inhibitory GABA agonists injected into the CM/Pf complex, revealing the reliance on the caudal intralaminar nuclei.

The SC has also been shown to be critical in transmitting short-latency visual information to dopamine neurons (Comoli et al., 2003), which are known for their phasic responses to unexpected, biologically-salient stimuli (Schultz et al., 1997). Notably, this activation causes phasic dopamine release in the striatum at approximately 110 ms following the event (Dommett et al., 2005), coinciding with the aforementioned dopamine-independent visually-evoked response in the spiny neurons. Moreover, there is evidence to suggest that both the striatal and dopaminergic responses to visual stimuli may be due to collaterals of the same SC projection (Coizet et al., 2007). Hence, there is a temporal convergence on striatal spiny neurons of excitatory thalamic input from the caudal intralaminar nuclei and dopamine from the SNc, informing the basal ganglia about the occurrence of salient events and providing neuromodulatory signals required for reinforcement learning (Wickens et al., 2003; Redgrave et al., 2008). The intralaminar nuclei also extensively innervate cholinergic interneurons in the striatum (Lapper and Bolam, 1992) and via these inputs mediate context-specific reinforcement learning mechanisms, specifically in dorsomedial striatum (Bradfield et al., 2013).

In addition to thalamic and dopamine projections, spiny neurons receive a vast input from almost all areas of the cerebral cortex, including collateral copies of ongoing motor commands from the motor cortices (Reiner et al., 2003). Changes in synaptic efficacy at these corticostriatal synapses are thought to underlie reinforcement learning, strengthening a motor representation by potentiating the efficacy of its corticostriatal projections. The cortical motor inputs, coupled with the convergence of sensory and dopamine signals described above, may present the necessary substrates to satisfy a three-factor learning rule required for long-term potentiation (LTP) at corticostriatal synapses (Reynolds and Wickens, 2002; Figure 1B). Pre-synaptic activity provided by the cortical inputs to be modified, in conjunction with thalamic activation from the intralaminar nuclei, may co-operate in some as yet unspecified manner to provide spiny neurons with the necessary signals and drive LTP by an appropriately timed phasic dopamine reinforcement signal. Phasic dopamine responses have also been shown to modulate corticostriatal plasticity in cholinergic interneurons (Suzuki et al., 2001; Reynolds and Wickens, 2004), however the association between this plasticity and learning has yet to be directly demonstrated in these interneurons, as it has with spiny neurons (Reynolds et al., 2001).

Thus far we have evidence of short-latency sensory and dopaminergic responses to salient visual stimuli that can simultaneously converge on spiny neurons via tecto-thalamo-striatal and tecto-nigro-striatal routes. Coupled with cortical motor signals, these components have the ability to modify corticostriatal weighting, a key control point in the cortico-basal ganglia loops. What could be the functional role of this system, which appears adept at responding quickly to salient sensory events, prior to the full processing that occurs post gaze orientation?

## Agency determination

A fundamental issue for an organism presented with an unexpected external event is to determine agency, i.e., if they were the agent causing the event, and which of their actions in particular was responsible. This is a problem in neural processing terms, as at any time during normal behavior a plethora of motor and sensory inputs are available, and only a small proportion are likely to relate to a recent action that caused a salient environmental event. This issue is critical in reinforcement learning in general, as an agent must be able to form action-outcome associations, in order to perform the same action in similar future circumstances and achieve the same desirable outcome.

The short-latency sensory and dopamine inputs are well suited for an instrumental role in a basal ganglia system that determines agency (Redgrave et al., 2011). To determine agency, the three factors of reinforcement learning previously described, including a short-latency signal via the intralaminar nuclei, are critical. Hence, a behavior causing an appropriately timed salient environmental event might be represented in the striatum by an excitatory cortical input representing the motor copy, followed by a convergence of excitatory thalamic input from the SC and dopaminergic input from the SNc. By creating the conditions for potentiation at corticostriatal synapses (Reynolds and Wickens, 2002), it has been proposed that this arrangement of inputs conveys to the organism that it was the agent that caused the sensory event by emitting a particular action (Redgrave et al., 2011). If, conversely, the convergence of thalamic and dopaminergic input arrived in the striatum in the absence of a preceding motor copy input, then this might convey to the organism that it was not the agent of that external environmental event.

In agency computations, short-latency signals are required so that processing can be performed before the relevant motor and sensory signals become contaminated with signals from the response to the sensory event, such as a gaze shift. Additionally, the shorter the latency, the greater the minimization of noise contributed by non-causal cortical signals. Repetition of potentiated actions by trial and error might strengthen the associations between the causal components of behavior and the outcome by dopamine-dependent LTP, and weaken non-causal coincidental behavior patterns via LTD (Reynolds and Wickens, 2002; Wickens et al., 2003; Redgrave et al., 2011). In this way agency determination would be resolved.

## Action selection

The existence of segregated, parallel loops involving the basal ganglia has been proposed as an elegant solution to the problem of behavioral selection (Redgrave et al., 1999, 2011). Selection becomes necessary due to the inherent conflicts arising when the large array of distributed sensory, cognitive and affective systems in the brain require access to the limited motor plant by which to initiate a movement response. Through its segregated loop architecture, the basal ganglia nuclei are able to process multiple competing actions simultaneously, and return neural activity associated with the action with the most desired outcome back to the sites of originating inputs. By removing inhibition of particular representations via its output nuclei, the basal ganglia can thus initiate a selected action (Chevalier and Deniau, 1990; Mink, 1996).

In recent years an appreciation of the importance of subcortical loops in action selection has been emerging (McHaffie et al., 2005; Redgrave et al., 2010). McHaffie et al. (2005) proposed the existence of at least three functionally segregated subcortical loop systems involving the SC and thalamic nuclei. Two of these loops are of particular relevance to this review (Figure 1A). First, the deep layer-rostral intralaminar thalamic loop, describing the connectivity from the deep SC layers, through the rostral intralaminar nuclei, and into the striatum. Second, the deep layer-caudal intralaminar thalamic loop, passing instead through the caudal intralaminar nuclei. These loops are considered segregated due to the different thalamic routes and also the nature of their thalamo-striatal synaptic contacts. Each exhibits different terminal arborization (Deschênes et al., 1995; Parent and Parent, 2005), synaptic contact points (Sidibé and Smith, 1999; Raju et al., 2006), and neuronal targets (Sidibé and Smith, 1996; Smith et al., 2004) across the dorsal and ventral striatum. In these cases, loops are formed by projections back to the SC from both major output nuclei of the basal ganglia, the SNr (Hopkins and Niessen, 1976; Redgrave et al., 1992) and the GPi (Takada et al., 1994).

With respect to the processing of salient visual events via the SC, there are at least two levels of selection operating. First, as the SC represents multiple stimuli in parallel, it must be able to selectively focus on a limited set of stimuli, and initiate a gaze shift towards a target. This selection case is likely handled via a non-intralaminar circuit from upper SC layers, through visual thalamic nuclei (e.g., lateral posterior nucleus), into the basal ganglia; and resulting in modulation of SC activity via disinhibition from the SNr, together with excitatory input from gaze centers (Takada et al., 1985; Hikosaka et al., 2006). Second, once visual selection of salient stimuli has occurred, the organism can select an appropriate early behavioral response—such as orient and approach, or withdraw and defend—based on the primitive stimuli features initially available. For instance, the SC is activated by dark “looming” stimuli in a number of species (Westby et al., 1990; Billington et al., 2011; Liu et al., 2011) and SC stimulation can elicit orientation and approach, or freezing and withdrawal responses, depending on the stimulation site (Sahibzada et al., 1986). Taking the rodent SC as a model, it mediates approach and withdrawal responses in distinct lateral and medial regions, respectively (Dean et al., 1986, 1989; Comoli et al., 2012), which are likely to have differential thalamic projections and potentially align with segregated tecto-thalamo-striatal intralaminar loops (Chevalier and Deniau, 1984). At the output of the basal ganglia, the nigro-tectal pathway is organized topographically so that the ventromedial SNr projects exclusively to the medial SC, while the dorsolateral SNr projects exclusively to the lateral SC (Redgrave et al., 1992; Comoli et al., 2012). Hence, it is possible that early selection of approach or withdrawal behavioral responses may be performed based on short-latency sensory inputs from the SC via subcortical loops through the intralaminar nuclei. Complete details of the functional roles of the SC-intralaminar loops await full elucidation.

### Competition between cortical and subcortical loops

**Figure 2:**
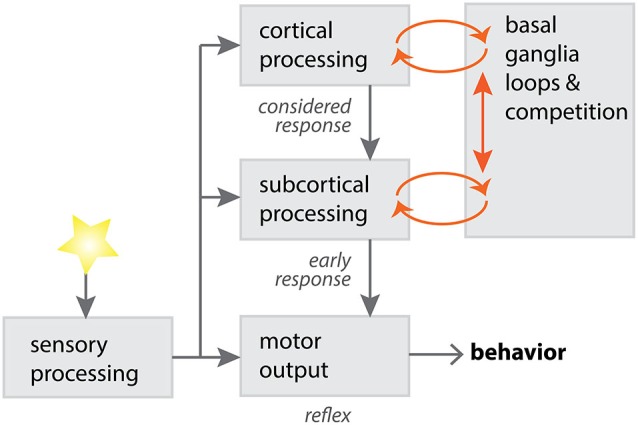
**A potential model for competition between cortical and subcortical processing, via loops through the basal ganglia**. Sensory input can be processed through different channels, with varying degrees of complexity in the processing operations. Subcortical processing through the basal ganglia, using relatively primitive sensorimotor systems, is adept at providing short-latency responses to salient stimuli (Schulz et al., 2009). Cortical processing through the basal ganglia is adept at longer-latency, more complex responses to stimuli. Cortical and subcortical requests for motor control can compete within the basal ganglia, with a “winner takes all” strategy necessary for effective behavioral selection (McHaffie et al., 2005; Redgrave et al., 2011). Diagram adapted with permission from P. Redgrave.

Competition between subcortical and cortical loops has been proposed (McHaffie et al., 2005), a competition biased by the anatomy of subcortical looped structures (Figure 2). For instance, there is a markedly greater density of thalamostriatal synapses on spiny neurons of the direct pathway (Sidibé and Smith, 1996; Huerta-Ocampo et al., 2013), whereas corticostriatal synapses are more evenly distributed between direct and indirect pathway neurons. As the direct pathway is thought to be responsible for action initiation (Albin et al., 1989; Alexander and Crutcher, 1990; Kravitz et al., 2010), subcortical thalamic loops have the potential to rapidly direct motor resources in response to a salient stimulus.

The deep layer-caudal intralaminar thalamic loop may also gain an advantage in motor control over cortical loops via its preferential innervation of cholinergic interneurons in the striatum. An important aspect of a selection system is to be able to quickly switch attentional and motor resources to a new salient stimulus—for instance one that might require fight or flight. To do this, all ongoing motor programs need to be rapidly inhibited. Short-latency SC-intralaminar signals would be optimally placed to perform this role, due to the SC’s access to salient sensory events, and the strong CM/Pf control of cholinergic striatal interneurons (Schulz et al., 2011).

In response to salient visual stimuli, tonically-active neurons in the striatum (putative cholinergic interneurons), exhibit a burst-pause sequence of activity (Morris et al., 2004; Goldberg and Reynolds, 2011; Schulz and Reynolds, 2013). This pattern requires an intact thalamic input from CM/Pf (Matsumoto et al., 2001), and can be induced by thalamic activation in an *in vitro* model (Ding et al., 2010). During the burst phase of the response, acetylcholine selectively enhances dendritic excitability of indirect pathway spiny neurons, with the net effect of biasing activation of indirect spiny neurons and increasing outflow from the indirect pathway (Shen et al., 2007). As the indirect pathway is generally thought to mediate motor suppression (Wichmann and DeLong, 1996), this circuit appears to be specialized for rapidly arresting ongoing motor activity in response to unexpected salient stimuli (Freeze et al., 2013).

In summary, the intralaminar nuclei, through their connections with the SC and basal ganglia, provide the striatum with short-latency access to salient stimuli important for behavioral learning and selection. Through connections with cholinergic interneurons, the caudal intralaminar pathway is able to exploit the basal ganglia motor control systems to suppress ongoing behavior and rapidly respond to a salient stimulus. The physiological advantages the subcortical loops have over cortical loops in the basal ganglia may explain how unpredicted sensory events, for example a bright light flash in the periphery, can rapidly command control of attentional and motor resources. This would appear to be evolutionarily advantageous to the survival of vertebrate organisms. Further, dysfunction of this circuit may be relevant to human psychiatric conditions characterized by hypervigilance, such as anxiety, or disordered agency, such as schizophrenia.
